# Using Multivariate Geostatistics to Assess Patterns of Spatial Dependence of Apparent Soil Electrical Conductivity and Selected Soil Properties

**DOI:** 10.1155/2014/712403

**Published:** 2014-12-31

**Authors:** Glécio Machado Siqueira, Jorge Dafonte Dafonte, Montserrat Valcárcel Armesto, Ênio Farias França e Silva

**Affiliations:** ^1^Centro de Ciências Agrárias e Ambientais, Universidade Federal do Maranhão, BR-222, Km 04, s/n, Boa Vista, 65500-000 Chapadinha, MA, Brazil; ^2^Departamento de Ingeniería Agroforestal, Escuela Politécnica Superior, Universidad de Santiago de Compostela, Campus Universitario, s/n, 27002 Lugo, Spain; ^3^Departamento de Engenharia Agrícola, Universidade Federal Rural de Pernambuco, Dois Irmãos, 52171-900 Recife, PE, Brazil

## Abstract

The apparent soil electrical conductivity (EC_a_) was continuously recorded in three successive dates using electromagnetic induction in horizontal (EC_a_-H) and vertical (EC_a_-V) dipole modes at a 6 ha plot located in Northwestern Spain. One of the EC_a_ data sets was used to devise an optimized sampling scheme consisting of 40 points. Soil was sampled at the 0.0–0.3 m depth, in these 40 points, and analyzed for sand, silt, and clay content; gravimetric water content; and electrical conductivity of saturated soil paste. Coefficients of correlation between EC_a_ and gravimetric soil water content (0.685 for EC_a_-V and 0.649 for EC_a_-H) were higher than those between EC_a_ and clay content (ranging from 0.197 to 0.495, when different EC_a_ recording dates were taken into account). Ordinary and universal kriging have been used to assess the patterns of spatial variability of the EC_a_ data sets recorded at successive dates and the analyzed soil properties. Ordinary and universal cokriging methods have improved the estimation of gravimetric soil water content using the data of EC_a_ as secondary variable with respect to the use of ordinary kriging.

## 1. Introduction

The quality of soil data collection for precision agriculture has a very important influence, since it has been found that acquisition of exhaustive information in this phase supports the use of geospatial technologies for the estimation of soil spatial variability and later on assists in the determination of “management units.” However, for assessing the soil spatial variability, a large number of samples are generally needed, which considerably increases costs of sampling and analysis. Notwithstanding, the sampling process can be improved, using soil variables that can be recorded or measured quickly, which can help in enhancing the estimation of other soil properties more difficult to measure.

The measurement of apparent soil electrical conductivity (EC_a_) allows the collection of information on the field and on the spatial distribution of other properties that are correlated. In accordance with Corwin and Rhoades [1] the main methods for the measurement of soil EC_a_ are contact and electromagnetic induction. McNeill [2], Sudduth et al. [3], Corwin and Lesch [4], and Kühn et al. [5] indicate that EC_a_ is mainly influenced by soil water content, texture, organic matter content, size and distribution of pores, salinity, cation exchange capacity, concentration of electrolytes dissolved in the soil solution, temperature, composition of soil colloids, and so on. Thus, the use of EC_a_ for soil classification allows recognition and delimitation of the physical, chemical, and biological soil properties that play an important role in agricultural production and environmental conservation. Thus, these data are essential for monitoring the temporal condition of the soil and application management processes [6]. Therefore, the EC_a_ parameter is used as an aid in precision agriculture, to promote the evaluation of the spatial variability of soil and the definition of management units.

The use of geostatistics has great advantages because it allows the study of the spatial variability of soil properties. Kriging is a geostatistical method that can be used to predict the value of soil properties in unsampled locations, favouring the application of differentiated soil management in precision agriculture. Several authors have devised soil sampling schemes directed by properties that directly or indirectly influence crop yield [7–10], and the success of this approach depends on the use of variables that are quickly and easily measured, such as EC_a_.

Based on the above rationale, the objectives of this work were as follows: (1) to analyze the spatial dependence of successive EC_a_ data sets, (2) to assess the spatial variability of soil texture attributes using a soil sampling scheme directed by soil EC_a_, and (3) to improve the estimation of the spatial variability of soil variables such as soil water content through multivariate geostatistics using EC_a_ as secondary information.

## 2. Material and Methods

### 2.1. Study Site

**Figure 1:**
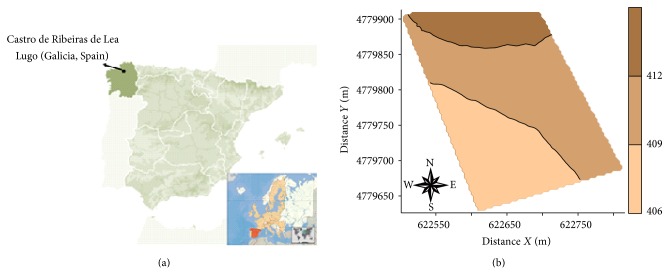
Geographical location of the study area (a). Field digital elevation model (b).

The experimental field is 6 ha in surface and it is located in Castro Ribeiras de Lea (Lugo, NW Spain). Geographic coordinates are 43°09′49′′N and 7°29′47′′ W, average elevation is 410 m, and mean slope is 2% (Figure 1).

The area where the field is located is considered to be representative of both the topographic patterns and the main soil type of the region “Terra Cha,” which is characterized by an extensive livestock production, on a landscape with seasonal conditions of hydromorphy, due to impeded drainage.

The crop succession of the experimental site was fallow-silage corn (*Zea mays* L.) under no-till farming during the study year. Previously, this site had been under pasture for silage production. Field data recording and soil sampling were performed in spring 2008.

The soil was classified as a Gleyic Cambisol [11], and it was developed over Tertiary-Quaternary sediments; the parent material from the Quaternary has high gravel content and it is underlain by clayey Tertiary sediments with low saturated hydraulic conductivity [12]. According to Neira Seijo [13] the soil profile of the studied field is represented by the sequence A_p_-B_w_-B_tg_, developed on successive sedimentary layers with heterogeneous soil particle size distribution (Table 1). The soil texture of the fine earth (<2.00 mm) was sandy-loam at the A_p_ horizon, sandy-clay-loam in B_w_ horizon, and clayey in the horizon B_tg_ and there was a general clay increase with soil depth. Moreover, the A_p_ and B_w_ horizons were characterized by a high content of gravel, attaining 37% and 45%, respectively. The organic matter content was rather high on the A_p_ horizon (5.05%) contrasting with the lower contents at the underlying horizons of the soil profile. The climate of the Terra Cha region is classified as maritime temperate climate (Cfb, according to Köppen), characterized by warm summers and no dry season; average annual rainfall is as high as 930 mm.

### 2.2. Apparent Soil Electrical Conductivity Measurements and Sampling Scheme

Apparent soil electrical conductivity (EC_a_) was measured using electromagnetic induction equipment EM38-DD [14]. This device consists of two integrated EM38 units oriented in the horizontal and vertical dipole positions, providing simultaneous measurements for the two dipoles modes; in the vertical dipole mode, the primary magnetic field is thought to effectively penetrate to a depth of about 1.5 m, while in the horizontal dipole position EM38-DD is thought to be effective for a shallower soil depth estimated at about 0.75 m [14].

**Figure 2:**
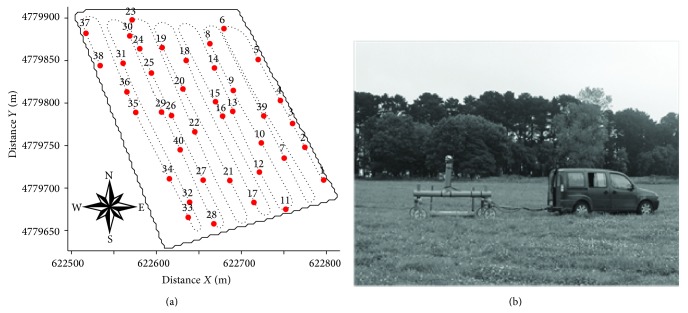
Scheme showing apparent electrical conductivity (EC_a_) continuously recorded (line) and the location of 40 soil sampling points (circles) on 23/6/2008 (a) and cart containing the EM38-DD equipment and GPS (b).

**Figure 3:**
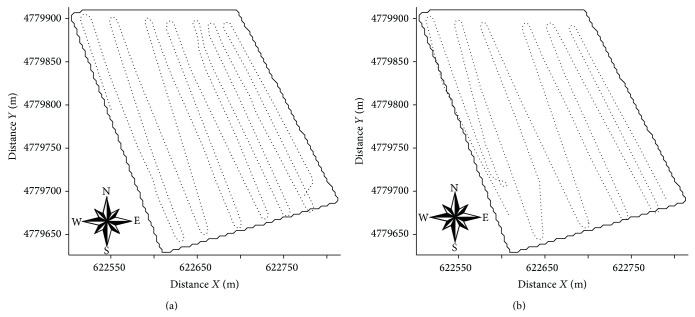
Scheme showing apparent electrical conductivity (EC_a_) recorded during 14/3/2008 (a) and 3/4/2008 (b).

To complete continuous record of the apparent soil electrical conductivity in horizontal dipole (EC_a_-H, mS m^−1^) and in vertical dipole (EC_a_-V, mS m^−1^) (Figures 2(a) and 3), the EM38-DD was installed in a car built with plastic materials (Figure 2(b)). In addition, GPS RTK was used for georeferencing the recorded measures.

The reference measurements of EC_a_-H and EC_a_-V were performed on 23/6/2008 at 1859 sampling points following the scheme presented in Figure 2(a). The soil sampling scheme was devised using the software tool ESAP-RSSD (response surface sampling design), based on a multiple linear regression model [7, 9]. This software aims to optimize the position of new sampling points considering apparent soil electrical conductivity (EC_a_) measured with horizontal (EC_a_-H) and vertical dipoles (EC_a_-V) (Figure 2(a)). The optimized soil sampling scheme consisted of 40 points. In addition continuous measurements of EC_a_-H and EC_a_-V were previously taken in the experimental field on 14/3/2008 and 3/4/2008, as shown in Figure 3. Note, however, that the schemes of the continuously recorded EC_a_ data sets taken in the three successive dates were different as shown in Figures 2(a), 3(a), and 3(b).

In the 40 points selected during the EC_a_ campaign of 23/6/2008, soil samples were taken at the 0.0–0.3 m depth with a manual soil probe. Soil texture, soil water content, and electrical conductivity of saturated paste extracts were determined using standard methods. Soil texture (clay, silt, and sand, in g kg^−1^) was determined by the sieve-pipette method, following Camargo et al. [15]; in this method a mixture of sodium hydroxide and sodium hexametaphosphate was used as chemical dispersant. The gravimetric soil water content (*θg*, %) was obtained after weighing the mass of the wet and dry sample, according to Camargo et al. [15]. To determine the electrical conductivity of soil saturated extracts (EC_e_), a mixture of soil and distilled water of 1 : 1 was prepared as proposed by USDA [16]; electrical conductivity measurements were performed using a conductivity meter ORION Model 122.

### 2.3. Statistical and Geostatistical Analysis

All the values were statistically analyzed using SPSS package 11.5 at 5% level of SNK (Student-Newman-Keuls) method ANOVA. The test of normality Kolmogorov-Smirnov was used to test the normality of data with probability of error 1% (*P* < 0.01). The correlation was calculated with the correlation coefficient of Pearson.

The analysis of the spatial variability of soil physical properties was conducted using the experimental variogram; the fitting of variogram model was performed using the method described by Vieira [17], based on cross-validation. Initial analysis showed that the variogram of any studied properties showed a trend, so the universal kriging was used in these cases, in which the residual variogram is required [18]. For those variables that showed no trend, ordinary kriging was used. The degree of spatial dependence (SD) was determined according to the following:

(1) $$\begin{array}{c} \text{SD}\left(\%\right)=\frac{C_{0}}{C_{0}+C_{1}}*100, \end{array}$$

where *C*_0_ is nugget effect and (*C*_0_ + *C*_1_) is the sill (*C*_0_ + *C*_1_) according to Cambardella et al. [19], which is considered as high (SD ≤ 25%), moderate (SD = 25–75%), and low (SD ≥ 75%).

Cross-variogram was used to study the spatial correlation between soil variables; when there was a trend in some of these variables, universal cokriging was used [18], instead of ordinary cokriging. The software used to perform ordinary kriging, universal kriging, and universal cokriging was Gstat [20]. In cokriging the covariance matrix must be positive and definite [18, 21–23]. The use of cokriging was used only for a couple of attributes that showed correlation coefficient values (|*r*|) greater than 0.5.

## 3. Results and Discussion

Statistical analysis of the data (Table 2) indicates that there is great variation between samples, in accordance with low (CV ≤ 12%) and middle (CV = 12–60%) variation coefficient values, by classification of Warrick and Nielsen [24]. It is verified that the apparent electrical conductivity of the soil (EC_a_) measurement with the horizontal dipole (EC_a_-H) has lower CV than the measurements with the vertical dipole (EC_a_-V). This fact can be explained because the vertical dipole mode explores a larger volume of soil than the horizontal dipole, and there is greater heterogeneity in those variables that affect the EC_a_ values, mainly clay content, organic material, water content in the soil, porosity, salinity, and so forth [2–5].

Only data EC_a_-V and EC_a_-H sampling in 23/6/2008 did not show differentiation by the average test (ANOVA) between the different sampling dates.

The values of the electrical conductivity of the saturation paste extract of the soil (EC_e_) are higher than the values of EC_a_-V and EC_a_-H; this fact is because EC_e_ is a parameter that depends on the content of anions and cations in the soil solution; the water content is homogeneous in all samples, because the sample is saturated with water, and the soil apparent electric conductivity values measured with the equipment EM38-DD (EC_a_-V and EC_a_-H) are very influenced by the soil water content [2, 3, 5]; and the water content in the soil is variable along the field.

EC_a_-V and EC_a_-H measured on several sampling dates (14/3/2008, 3/4/2008, and 23/6/2008) showed lognormal distribution (Table 2). Other attributes studied showed normal frequency distribution (EC_e_, clay, silt, sand, and soil water content).

In the geostatistical analysis, lognormal transformation was used for properties that showed lognormal distribution. The highest values of coefficient of correlation between EC_a_ variables and clay and silt content are on the first measurement date (14/3/2008); on this date the soil moisture is lower coincided with data of precipitation and evapotranspiration (Table 3). Grandjean et al. [25] describe that soil with moisture lower is ideal for characterization of soil bulk density and of soil texture, using measurements of electrical conductivity.

The coefficient of correlation values between the apparent soil electrical conductivity (EC_a_-V and EC_a_-H) measurement on several sampling dates (14/3/2008, 3/4/2008, and 23/6/2008) presented moderate positive correlation coefficient values (0.5 ≤ *r* < 0.8).

The coefficient of correlation between EC_a_-V_23/6/2008_ × *θg* (*r* = 0.685) and EC_a_-H_23/6/2008_ × *θg* (*r* = 0.648) was moderately positive (0.5 ≤ *r* < 0.8), confirming the correlation between the values of EC_a_-V and EC_a_-H and the water content in soil, because according to Grandjean et al. [25], under wet conditions, electrical conductivity measurements are dominated by the effect of water content, which tends to hide the influence of the other factors.

**Figure 4:**
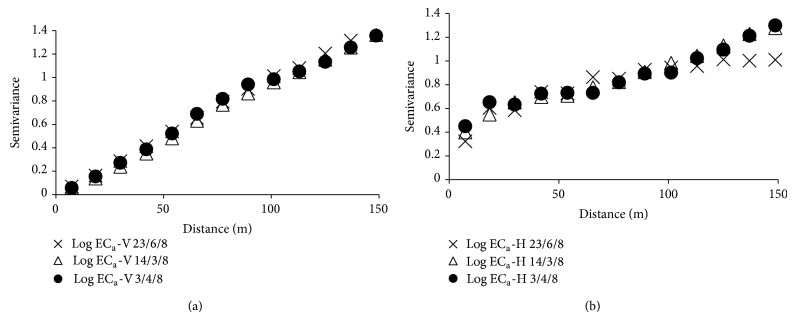
Standardized sample semivariogram for log EC_a_-V (a) and log EC_a_-H (b) recorded during three successive dates.

The values of log EC_a_-V are affected by the groundwater level, so the variogram follows the trend in the ground water level (Figure 4). As can be seen on standardized variograms with the value of the sample variance, data from EC_a_-V measurement on 14/3/2008 and 3/4/2008 present a trend, following the same pattern of the digital elevation map of the area (Figure 1(b)). Analyzing standardized variograms for log EC_a_-H data can be seen that only shows trend for the variogram of 14/3/2008 and 3/4/2008, but not for the variogram of 23/6/2008; on this date the water table was probably located below the depth of soil investigated with the horizontal dipole mode.

Corwin and Lesch [4] found higher values of correlation between data from log EC_a_-V and log EC_a_-H and electrical conductivity of the saturation extract (EC_e_) and clay content, but lower than those found for log EC_a_-V and log EC_a_-H and the water content in soil. Martínez and Vanderlinden [26] described a higher correlation between EC_a_ and water content in loamy soils, while in clay soils the correlation was lower. The correlation coefficients for EC_e_, silt, and clay with the log EC_a_-H are greater than with the log-EC_a_-V.

In order to improve the correlation between the values of EC_a_-V and EC_a_-H with clay content, soil water content should be as homogeneous as possible within the study area, better if its value is closer to field capacity and unlike the water table is as low as possible, so the best time to take measurements under these conditions would be in the autumn, when there was heavy rainfall, although under these conditions the water table probably would not have ascended enough to be close to the surface.

The initial geostatistical data analysis showed that the physical properties of the soil (clay, silt, sand, and gravimetric water content) showed no trend, then being possible the estimate of the variable using the original data with ordinary kriging. Moreover, EC_e_ data and apparent electrical conductivity of the soil (EC_a_-V and EC_a_-H) on several sampling dates show trend in Figure 3; the semivariance value is not stabilized around variance value of data, and the universal kriging was used to construct the maps of spatial variability of these variables.

The fitted variogram parameters (Table 5) show that the spherical model was the fitted model to the properties under study, according to Cambardella et al. [19], Goovaerts [18], Vieira [17], and Siqueira et al. [27] describing this model; it is usually best fitted to the properties of soil and plant. All attributes had low values of nugget effect (*C*_0_). Range values (a) varied from 40.00 m (log EC_a_-H Residual_3/4/2008_) to 130.00 m (clay, silt, and soil water content). The degree of spatial dependence between samples was high (SD ≤ 25.00%) across the study, the exception being log EC_a_-H Residual_14/3/2008_ which presented a moderate degree of spatial dependence (SD = 31.67%).

**Figure 5:**
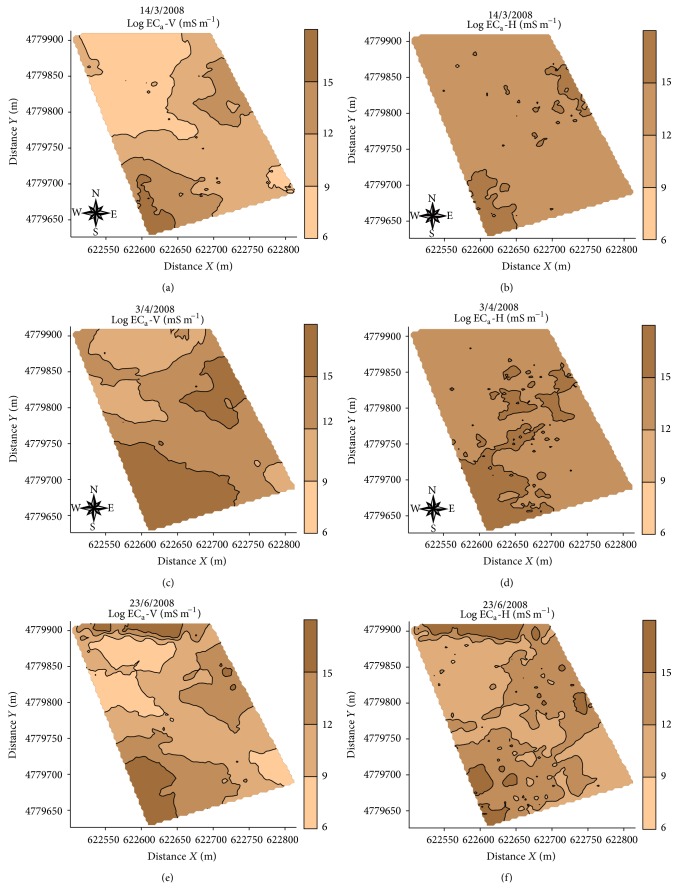
Kriging maps of apparent soil electrical conductivity (EC_a_-V and EC_a_-H) from the continuous records made in 14/3/2008, 3/4/2008, and 23/6/2008.

The spatial variability maps obtained with universal kriging (Figure 5) show that there is a similarity between the maps EC_a_-V (Figures 5(a), 5(c), and 5(e)) and EC_a_-H (Figures 5(b), 5(d), and 5(f)) on several sampling dates, with further differentiation of maps of EC_a_-V and EC_a_-H on the measured data on 23/6/2008 (Figures 5(e) and 5(f)) when the water table level was lower.

**Figure 6:**
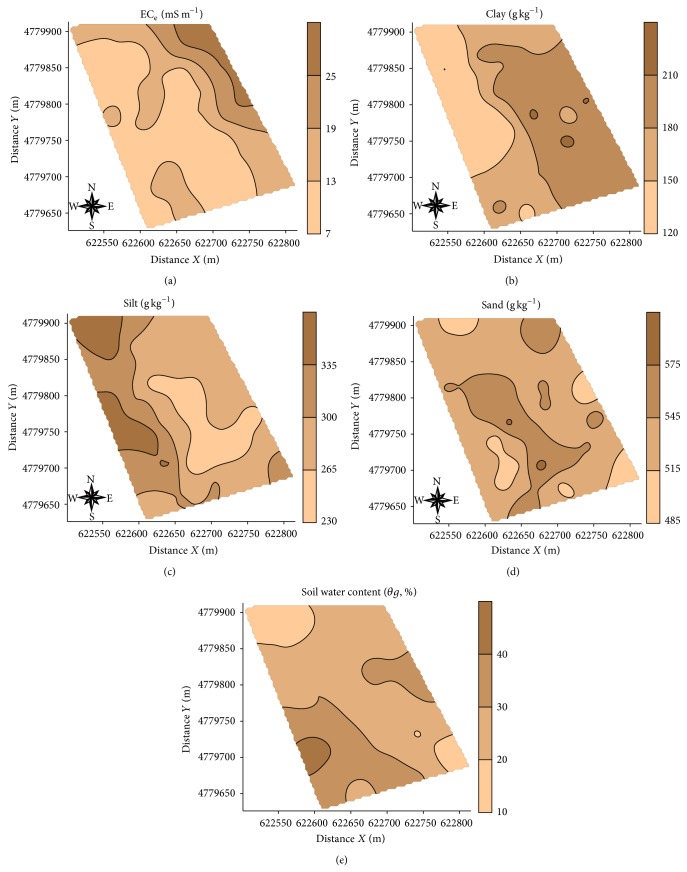
Kriging maps of the soil properties analyzed.

It is observed that the maps of EC_a_-V and EC_a_-H (Figure 5) and the map of the water content in soil (Figure 6(e)) obtained with ordinary kriging (Figure 6(e)) look similar, following the same pattern of digital elevation model (Figure 1(b)).

The map of the electrical conductivity of the saturation extract (EC_e_, Figure 6(a)) shows inverse behaviour to maps EC_a_-V and EC_a_-H (Figure 5). Moreover, the map of spatial variability of clay content in the study area (Figure 6(b)) shows no similarity to maps EC_a_-V and EC_a_-H (Figure 5); this fact is also repeated with silt (Figure 6(c)) and sand (Figure 6(d)).

Table 6 presents the fitting parameters cross-variogram between *θg* × EC_a_-V (*r* = 0.685) and *θg* × EC_a_-H (*r* = 0.648). The cross-variograms were fitted to a spherical model with the same range compared to single variograms to obtain a linear model coregionalization (Table 6).

**Figure 7:**
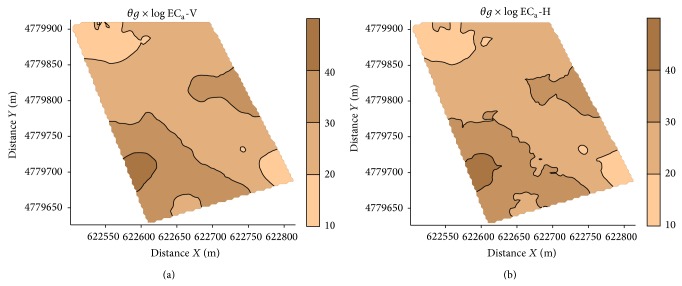
Map of gravimetric water content, *θg*, obtained by the universal cokriging.

The spatial variability maps constructed using ordinary and universal cokriging (Figure 7) demonstrate that the use of the soil apparent electrical conductivity measured by electromagnetic induction (EC_a_-V and EC_a_-H) on 23/6/2008 was a secondary variable that improves the estimation of the soil water content using cokriging. This improvement in the estimation of *θg* can be observed in Table 7, where it showed an increase in the value of the correlation coefficient between the measured and the estimated values from cross-validation using ordinary cokriging with log-EC_a_-V (0.746) and with log-EC_a_-H (0.756) as secondary variables with respect to use of ordinary kriging (0.637). Moreover, in the case of the soil water content map obtained with ordinary cokriging using as secondary data EC_a_-H (Figure 7(b)) is less smooth than the map obtained with ordinary kriging (Figure 6(e)).

## 4. Conclusions

When taking into account all the soil properties studied, EC_a_ and gravimetric soil water content measured at the same date, that is, 23/6/2008, showed the highest coefficients of correlation Table 4. Moreover, EC_a_ showed higher coefficients of correlation to clay and silt content than to silt content, and the strength of the correlation was higher for the first EC_a_ recording date, that is, 14/3/2008, when the soil moisture was lower. Thus, coefficient of correlation of EC_a_ with silt and clay content showed a trend to increase the soil moisture decreased; this result suggests the usefulness of recording EC_a_ on successive dates with different soil water contents.

The spatial patterns of spatial variability of the logarithmic values of apparent soil electrical conductivity (EC_a_) and the electrical conductivity of the soil saturated paste (EC_e_) were modeled by universal kriging, whereas those of sand, clay, silt, and gravimetric water content were modeled by ordinary kriging. The use of cokriging with EC_a_ data as secondary variable improved the estimation of the gravimetric soil water content with respect to the use of kriging.

## Figures and Tables

**Table 1:** Soil texture data for a representative profile of the study area.

Horizon	Depth (m)	Organic matter	Clay	Silt	Sand	Gravel
		g dm^−3^	g kg^−1^			
A_p_	0.0–0.35	50.50	175	191	634	370
B_w_	0.35–0.70	7.20	192	207	591	448
B_tg_	>0.70	2.60	479	280	241	—

**Table 2:** Statistical parameters of the continuously recorded EC_a_ data sets and the soil properties analyzed.

Date	Variable	Unit	*N*	Min.	Max.	Mean ± SD	Variance	CV	Skew	Kurt	*D*
14/3/2008	EC_a_-V	mS m^−1^	1887	5.75	18.38	10.48 ± 1.19	1.42	11.35	0.527	0.124	0.045Ln
14/3/2008	EC_a_-H	mS m^−1^	1887	9.25	19.00	14.1 ± 0.77	0.60	5.46	0.065	1.810	0.040Ln
3/4/2008	EC_a_-V	mS m^−1^	1871	9.63	20.50	14.04 ± 2.15	4.64	15.31	0.662	0.083	0.073Ln
3/4/2008	EC_a_-H	mS m^−1^	1871	6.63	19.50	14.59 ± 0.77	0.60	5.28	0.160	10.51	0.095Ln
23/6/2008	EC_a_-V	mS m^−1^	1886	4.13	20.13	11.21 ± 2.47^*^	6.12	22.03	0.485	−0.243	0.071Ln
23/6/2008	EC_a_-H	mS m^−1^	1886	6.63	20.00	12.12 ± 1.79^*^	3.22	14.77	0.839	1.285	0.092Ln
23/6/2008	CE_e_	mS m^−1^	40	7.00	28.00	13.82 ± 5.09	25.94	36.83	1.200	1.008	0.159n
23/6/2008	Clay	g kg^−1^	40	119.00	220.00	168.37 ± 30.92	956.54	18.36	−0.190	−1.321	0.153n
23/6/2008	Silt	g kg^−1^	40	233.00	357.00	296.25 ± 35.06	1229.73	11.83	0.149	−1.041	0.098n
23/6/2008	Sand	g kg^−1^	40	487.00	586.00	535.37 ± 22.53	507.72	4.21	0.055	−0.270	0.069n
23/6/2008	*θg*	%	38	13.41	45.67	26.74 ± 6.81	49.50	25.47	−2.904	6.510	0.085n

*N*: number of measurements; Min.: minimum value; Max.: maximum value; Mean ± SD: mean ± standard deviation; CV: coefficient of variation (%); Skew: skewness; Kurt: kurtosis; and *D*: normality of the data for test of Kolmogorov-Smirnov (*P* < 0.01, n: normality, and Ln: log normality). ^*^Nonsignificant at 5% level of ANOVA (SNK).

**Table 3:** Precipitation and reference evapotranspiration between successive dates, in which apparent soil electrical conductivity (CE_a_-V and CE_a_-H) was recorded.

Period	Precipitation (mm)	Reference evapotranspiration (mm)
15/2/2008–14/3/2008	52.6	38.0
14/3/2008–3/4/2008	80.4	37.1
3/4/2008–23/6/2008	397.8	214.6

**Table 4:** Linear correlation matrix between the continuously recorded EC_a_ data sets and the soil properties analyzed.

		14/03/2008		03/04/2008		23/06/2008						
		Log EC_a_-V	Log EC_a_-H	Log EC_a_-V	Log EC_a_-H	Log EC_a_-V	Log EC_a_-H	EC_e_	Clay	Silt	Sand	*θg*
14/03/2008	Log EC_a_-V	1.000										
14/03/2008	Log EC_a_-H	0.780	1.000									
03/04/2008	Log EC_a_-V	0.972	0.759	1.000								
03/04/2008	Log EC_a_-H	0.724	0.796	0.792	1.000							
23/06/2008	Log EC_a_-V	0.861	0.729	0.855	0.688	1.000						
23/06/2008	Log EC_a_-H	0.541	0.644	0.515	0.569	0.751	1.000					
23/06/2008	EC_e_	0.127	0.185	−0.012	−0.141	0.156	0.224	1.000				
23/06/2008	Clay	0.344	0.495	0.252	0.197	0.253	0.346	0.221	1.000			
23/06/2008	Silt	−0.247	−0.423	−0.172	−0.216	−0.137	−0.228	−0.145	−0.773	1.000		
23/06/2008	Sand	−0.086	−0.012	−0.076	0.065	−0.133	−0.119	−0.076	−0.170	−0.494	1.000	
23/06/2008	*θg*	∗	∗	∗	∗	0.685	0.648	0.005	0.221	−0.145	−0.075	1.000

^*^Values were excluded in linear correlation analysis because the soil water content was only measured on 23/6/2006 and for this reason the coefficients of correlation were not calculated between soil water content and apparent electrical conductivity of the soil (EC_a_-V and EC_a_-H) for other sampling dates (14/3/2008 and 03/4/2008).

^**^To correlate the measurement of log EC_a_-V and log EC_a_-H for the measurement dates (14/3/2008 and 3/4/2008) with the measurements made on 23/6/2008, it was necessary to estimate the measurements of log EC_a_, EC_e_, clay, silt, and sand content using kriging in locations measured on 23/6/2008.

**Table 5:** Fitted semivariogram parameters and respective models of the continuously recorded EC_a_ data sets and the soil properties analyzed.

Date	Variable	Geostatistical method	Model	*C*_0_	*C*_1_	*a*	SD
14/03/2009	Log EC_a_-V residual	UK	Spherical	0.0001	3.14	105.00	0.00
	Log EC_a_-H residual	UK	Spherical	0.14	0.302	44.00	31.67

03/04/2008	Log EC_a_-V residual	UK	Spherical	0.00	5.10	145.00	0.00
	Log EC_a_-H residual	UK	Spherical	0.10	0.32	40.00	23.80

23/06/2008	Log EC_a_-V residual	UK	Spherical	0.001	0.01	130.00	9.09
	Log EC_a_-H residual	UK	Spherical	0.001	0.005	130.00	1.96
	EC_e_ residual	UK	Spherical	0.00025	0.0018	100.00	9.09
	Clay	OK	Spherical	0.001	1060.00	130.00	0.00
	Silt	OK	Spherical	0.00	1400.00	130.00	0.00
	Sand	OK	Spherical	0.00	510.00	70.00	0.00
	*θg*	OK	Spherical	0.001	60.00	130.00	0.00

UK: universal kriging; OK: ordinary kriging; *C*_0_: nugget effect; *C*_1_: structural variance; *a*: range (m); and SD: spatial dependence (%).

**Table 6:** Fitted cross-semivariogram models and respective parameters between gravimetric water content (principal variable) and log⁡EC_a_ (secondary variable).

	Variable	Geostatistical method	Model	*C*_0_	*C*_1_	*a* (m)
23/6/2008	*θg* × log⁡EC_a_-V	Universal cokriging	Spherical	1.00	20.00	130.00
23/6/2008	*θg* × log⁡EC_a_-H	Ordinary cokriging	Spherical	0.00	15.00	130.00

*C*_0_: nugget effect; *C*_1_: structural variance; and *a*: range (m).

**Table 7:** Correlation coefficients between measured gravimetric water content and data estimated by kriging and cokriging.

*θg* (ordinary kriging)	0.637	*θg* × log⁡EC_a_-V (universal cokriging)	0.746
		*θg* × log⁡EC_a_-H (ordinary cokriging)	0.756

## Abbreviations

EC_a_: Apparent soil electrical conductivity
EC_a_-V: CE_a_ in vertical dipole
EC_a_-H: CE_a_ in horizontal dipole
EC_e_: Electrical conductivity of soil saturation extract.
